# The development and initial validation of the Russian version of the BASIS-24

**DOI:** 10.1186/s13722-022-00343-0

**Published:** 2022-11-26

**Authors:** Lynn M. Madden, Scott O. Farnum, Daniel J. Bromberg, Declan T. Barry, Alyona Mazhnaya, Tetiana Fomenko, Anna Meteliuk, Ruthanne Marcus, Julia Rozanova, Iurii Poklad, Sergii Dvoriak, Frederick L. Altice

**Affiliations:** 1 APT Foundation, Inc, 1 Long Wharf Drive, Suite 321, New Haven, CT 06511 USA; 2 Department of Psychiatry, Yale University School of Medicine, 300 George Street, Suite 901, New Haven, CT 06510 USA; 3 Yale School of Public Health, Laboratory of Epidemiology and Public Health, 60 College St, New Haven, CT 06510 USA; 4 Section of Infectious Diseases, Yale University School of Medicine, 333 Cedar Street, New Haven, CT 06510 USA; 5 ICF Alliance for Public Health, 24 Bulvarno-Kudriavska Street, Kyiv, 01601 Ukraine; 6 Chernihiv Regional Narcological Dispensary, 3 Shchorsa Street, Chernihiv, 14005 Ukraine; 7 European Institute of Public Health Policy, 1 Malopidvalna Street, Office 10, Kiev, 01054 Ukraine; 8 Centre of Excellence On Research in AIDS (CERiA), University of Malaya, Malaysia Level 17, Wisma R&DJalan Pantai Baharu, 59990 Kuala Lumpur, Malaysia; 9 Child Study Center, Yale University School of Medicine, 230 S Frontage Road, New Haven, CT 06519 USA; 10 School of Public Health, National University of Kyiv-Mohyla Academy, Hryhoriya Skovorody Street, Kiev, 2 04655 Ukraine

**Keywords:** Medications for Opioid Use Disorder (MOUD), Eastern Europe and Central Asia (EECA), HIV, BASIS-24-R, PWID, Implementation Science, Validation

## Abstract

**Background:**

Efficient and linguistically appropriate instruments are needed to assess response to addiction treatment, including severity of addiction/mental health status. This is critical for Russian-speaking persons in Eastern Europe and Central Asia (EECA) where Medications for Opioid Use Disorder (MOUD) remain underscaled to address expanding and intertwined opioid, HIV, HCV and tuberculosis epidemics. We developed and conducted a pilot validation of a Russian version of the 24-item Behavior and Symptom Identification Scale (BASIS-24), an addiction/mental health severity instrument with six subscales, previously validated in English.

**Methods:**

Using the Mapi approach, we reviewed, translated, and back-translated the content to Russian, pilot-tested the Russian-version (BASIS-24-R) among new MOUD patients in Ukraine (N = 283). For a subset of patients (n = 44), test-rest was performed 48 h after admission to reassess reliability of BASIS-24-R. Exploratory principal component analysis (PCA) assessed underlying structure of BASIS-24-R.

**Results:**

Cronbach alpha coefficients for overall BASIS-24-R and 5 subscales exceeded 0.65; coefficient for Relationship subscale was 0.42. The Pearson correlation coefficients for overall score and all subscales on the BASIS-24-R exceeded 0.8. Each item loaded onto factors that corresponded with English BASIS-24 subscales ≥ 0.4 in PCA.

**Conclusion:**

Initial version of BASIS-24-R appears statistically valid in Russian. Use of the BASIS-24-R has potential to guide MOUD treatment delivery in the EECA region and help to align addiction treatment with HIV prevention goals in a region where HIV is concentrated in people who inject opioids and where healthcare professionals have not traditionally perceived MOUD as effective treatment, particularly for those with mental health co-morbidities.

## Introduction

The opioid epidemic in the EECA region is among the highest globally [15] and is intertwined with some of the highest rates of HIV, HCV, tuberculosis and incarceration [5, 25], resulting in an urgent public health crisis. Key to controlling these epidemics is the scale-up of Medications for Opioid Use Disorder (MOUD), i.e., methadone (MMT) or buprenorphine (BMT) maintenance therapy among persons who inject drugs (PWID). MOUD is not only one of the most effective strategies for controlling HIV [28], HCV [40] and tuberculosis [38], but when scaled adequately it can reduce population-based transmission and mortality in the EECA region [3, 36], 48]. Coverage levels of MOUD in this region, however, are extraordinarily low, often available as pilot programs or not present at all [26].

MOUD was introduced into the EECA region not for the treatment of opioid use disorder, but rather for the prevention of HIV [27]. In this region, due primarily to external pressure from the Russian Federation where MOUD is banned, substance use treatment specialists (i.e., “Narcologists”) are primarily responsible for prescribing MOUD. Scale-up of MOUD has been limited because, despite all the evidence of the effectiveness of MOUD to treat opioid use disorder, it is often viewed as ineffective for treating opioid use disorder [41, 43, 50], leaving many substance use treatment specialists to focus on inpatient “detox” followed by residential treatment—a strategy that has not been found to be effective [23] for the treatment of opioid use disorder nor for the prevention and/or treatment of HIV.

In addition, concerns about the complexity of patients with opioid use disorder, including psychiatric co-morbidities may prevent providers from offering MOUD [11, 29]. Brief, culturally appropriate screening instruments would allow providers in the EECA region to assess and address the complexity of patients receiving MOUD to improve treatment retention [1] as well as improve treatment access as processes and programs can be better tailored to meet their clinical needs. Few validated psychometric instruments, however, exist in Russian for routine use in medical practice [6]. The countries of EECA share language heritage from the former Soviet Union and Russian is used in many places as a first or second language. Overall, Russian is spoken by more than 150 million people globally as a native language [21], and by over 260 million total speakers. The aim of this study was to develop and perform an initial validation of the Russian version of the BASIS-24, a 24-item scale that measures patient symptom severity across six domains that was originally developed and validated in English and has also been validated in Spanish [14, 16–19]. Having a validated instrument in Russian allows comparison of patient progress in treatment across important functional and mental health domains and could provide guidance to clinicians to substantially advance the promotion and the efficacy of MOUD as an effective treatment for opioid use disorder for Russian-speaking populations.

## Methods

### Measures

The BASIS-24© consists of 24 items with an overall scale score and 6 subscales assessing: depression (6 items), interpersonal relationships (5 items), self-harm (2 items), emotional lability (3 items), psychotic symptoms (4 items), and alcohol/drug use (4 items). Each item is rated on a five-point scale with response options indicating level of severity or frequency of occurrence during the past week.

The methodology for developing and evaluating the initial validation was based on the Mapi Institute model for translating clinical outcome measures [2], which was also used in a previous validation study of the BASIS-24 in Spanish [16]. The Mapi method is intended to produce a version of an instrument that embodies the conceptual framework of the original instrument that can allow data comparison across languages and countries. The steps of the Mapi method for linguistic validation of healthcare instruments are: (1) forward translation; (2) reconciliation; (3) back translation; and (4) pilot testing. Consistent with the steps of the Mapi method, the BASIS-24 was first translated from English to Russian by 2 certified translators, reconciled among the study group by discussion, and then back-translated for cultural applicability by 2 native Russian speaking members of an addiction treatment research team in Ukraine fluent in English and with MPH degrees from US institutions. After agreement in the translation/back-translation process [10], a consensus version of the instrument was field tested in November 2014 to ensure understanding.

### Recruitment and testing

As part of an implementation science study using NIATx [12, 22, 33, 44] to scale-up MOUD across Ukraine, a subset of 10 addiction treatment specialists (representing 10 sites) agreed to administer the Russian-version of BASIS-24 (BASIS-24-R) to consecutive patients newly enrolling in MOUD. The sites included two dedicated addiction treatment facilities, two AIDS Centers and 2 TB Centers that provided integrated care, and four general hospitals—all MOUD sites were run by addiction treatment specialists (Narcologists) in urban areas. All new admissions between 11/1/14 and 5/16/16 were assessed with the BASIS-24-R. In addition to the 24 scale score items of the BASIS-24-R, the survey included items that measured self-reported socio-demographics (see Table 2). The instrument was translated and back-translated in Ukrainian, however none of the participants requested the instrument in Ukrainian though it was offered.

The target enrollment was 240 patients to ensure sufficient sample size for a 10:1 ratio of responses to item. Overall, 293 initiated the assessment; individuals who did not complete two or more items on the survey (N = 10) were removed, leaving 283 for the final analysis. To ascertain test–retest reliability, a pilot test subset of 15% (44) of the original participants then were retested within 48 h of the first survey.

### Analysis

The suitability of BASIS-24-R was suggested by the: (1) sample size (more than 10 participants per item); (2) Kaiser-Myer-Olkin Ration (which indicates how much of the variance or variability is common to a set of variables; minimum set at 0.6); and (3) Bartlett’s test of sphericity (which examines variability among different combinations of data; alpha level set at < 0.5) [31, 46]. Reliability was tested using two common approaches. First, internal reliability (i.e., the degree to which items grouped together) was tested for each subscale using Cronbach’s alpha (minimum set at 0.7 for significance). Second, test–retest reliability was calculated using Pearson’s correlation coefficient (with a minimum set at |0.5| for significance). We performed an exploratory principal components analysis (PCA) on the 24 scale score items to investigate whether there was an underlying structure in the pattern of correlations using the guidelines put forth by Tinsley and Tinsley [49]. A minimum loading factor of 0.4 on any given subscale was considered significant. All analyses were conducted in SPSS and RStudio [4].

#### Results

Table 1 presents the demographic characteristics with majority of respondents being between 25 and 44 years old; 83% were male. The majority of the sample graduated from high school, with 49% having some form of college education and 56% of the sample being unemployed. Table 2 summarizes findings related to BASIS-24-R internal reliability and test–retest reliability.

**Table 1:** Characteristics of the sample completing the BASIS-24-R (N = 283)

Characteristics	N	%
Mean age, years (S.D.)		
Age categories (years)		
18–24	14	4.9
25–34	130	45.9
35–44	110	38.9
45–54	17	6.0
55–64	0	0.0
65 +	1	0.4
No response	11	3.9
Sex		
Male	235	83.0
Female	47	16.6
No response	1	0.4
Marital status		
Never married	89	31.4
Married	101	35.7
Separated	28	9.9
Divorced	51	18.0
Widowed	11	3.9
No response	3	1.1
Education		
8th grade or less	16	5.7
Some high school	108	38.2
High school grad/GED	18	6.4
Some college	101	35.7
4-Year graduate	37	13.1
No response	3	1.1
Employed past 30 days		
No	159	56.2
Yes, 1–30 h	44	15.5
Yes, 11–30 h	31	11.0
Yes, over 30 h	49	17.3
Unpaid volunteer	16	5.6
Unpaid student	6	2.0

**Table 2:** Internal consistency and test–retest reliability of the Russian Version of the BASIS-24

Subscale	Number of Items	Internal consistency reliability of α^a^ (n = 283)	Test–retest reliability ^b^ (n = 44)
Depression/functioning	6	0.880	0.953
Relationships	5	0.422	0.846
Self harm	2	0.717	0.959
Emotional lability	3	0.835	0.915
Psychosis	4	0.727	0.968
Substance abuse	4	0.654	0.930
Overall	24	0.889	0.976

^a^Cronbach’s alpha; minimum set at 0.7 for significance

^b^Pearson correlation coefficient; minimum set at |0.5| for statistical significance

In terms of internal consistency, all of the scales had alpha coefficients of 0.654 or above, with the exception of the Relationship subscale (α = 0.422). The overall scale had a Cronbach alpha of 0.889. The Pearson (Test–Retest results) correlations were 0.846 or above for subscales and 0.976 for the overall scale.

Table 3 summarizes the factor loadings of each question, which ranged from 0.402 to 0.845. All questions loaded at 0.4 or above on the expected subscale. Four questions (9, 6, 21 and 22) also loaded onto another subscale, as part of the sensitivity analysis. In each case, the higher factor loading occurred on the expected BASIS-24 subscale. For example, Question 9 loaded onto the alternative subscale at 0.402 and loaded onto the expected Depression and Functioning subscale at 0.563.

**Table 3:** Exploratory Factor Analysis for Each Subscale (N = 283)

Item	M (SD)	Factor Loading (.4 or above)
Subscale: depression & functioning		
Q1	1.88 (1.324)	.768
Q2	2.09 (1.253)	.777
Q3	1.54 (1.322)	.845
Q9	1.88 (1.261)	.563 (.402)
Q10	2.04 (1.243)	.717
Q12	2.02 (1.321)	.764
Subscale: interpersonal relationships		
Q4	2.08 (1.109)	0.495
Q5	2.47 (0.928)	0.817
Q6	2.69 (1.067)	0.563 (.474)
Q7	2.55 (1.239)	0.713
Q8	2.03 (1.266)	0.742
Subscale: self harm		
Q11	0.92 (1.138)	0.699
Q20	0.71 (1.026)	0.729
Subscale: emotional lability		
Q13	1.98 (1.313)	0.542
Q18	2.11 (1.195)	0.734
Q19	1.99 (1.172)	0.664
Subscale: psychosis		
Q14	0.43 (.839)	0.634
Q15	0.24 (.632)	0.717
Q16	1.09 (1.134)	0.811
Q17	1.12 (1.112)	0.637
Subscale: alcohol & drug use		
Q21	2.64 (1.422)	0.546 (0.446)
Q22	2.17 (1.123)	0.468 (0.444)
Q23	2.10 (1.338)	0.815
Q24	2.30 (1.185)	0.549

## Discussion

Findings from this study demonstrate high internal consistency and re-test reliability for the BASIS-24-R, supporting its potential for use in the region. The continually high rate of new HIV infections in the region requires urgency in better understanding the treatment needs for PWID who remain at the center of HIV transmission in the EECA region and for whom little gains have been made in HIV transmission and mortality [24]. Currently there are no psychometric tools validated in Russian that can be used in routine clinical practice to guide substance use treatment experts, making it difficult to understand the complexities of the growing number of people who inject opioids, including their psychiatric and psychological co-morbidities. This will be especially true as MOUD is increasingly delivered by experts in treatment of HIV or TB in integrated care settings [7, 35] or by primary care physicians [34, 36, 37] who have little specialty training in substance use treatment. The BASIS-24-R can benefit both the needs of clinicians to understand and successfully treat those with OUD as well as patients who may experience improved access to care, as well as improved care pathways as their co-morbid needs are met. Though the BASIS-24-R was validated in outpatient MOUD settings, both the English and Spanish versions included inpatients in their samples, suggesting that the BASIS-24-R may have utility in the detox settings that are common in the EECA region.

In the EECA region where the prevalence of opioid use disorder is high and contributes the most to elevated HIV morbidity and mortality, brief and validated tools are needed to assess progress along the treatment continuum and the validated BASIS-24-R instrument provides a new tool. Multiple studies from this region affirm the notion that MOUD is effective in preventing HIV and therefore potentially reinforce the idea that MOUD should not be the treatment of last resort in treating OUD [8, 9, 13, 30, 32, 45]. Yet the prescribers of MOUD, addiction treatment specialists and other clinicians, would benefit if they could document improvements in health using a brief tool which would not only document changes, but identify key priorities areas (e.g., mental health, relationships, etc.) where they could focus their efforts on guiding their patients through recovery.

Having this tool could potentially reduce barriers to MOUD scale-up, a strategy that is crucial to prevent HIV-related transmission and reduce mortality in the region [48], including in prisons [5, 15, 47]. The BASIS-24-R, by virtue of pilot validation in the EECA, has the promise of providing a brief and useful tool to assess patients and provide the additional services that might benefit patients. It also overcomes the “not invented here” perception where research illustrates a strong bias against ideas from the outside [39], especially with regard to MOUD in EECA [41, 41–43, 43].

Findings from this study demonstrate high internal consistency and re-test reliability for the BASIS-24-R, supporting its potential for use in the Russian-speaking region. The continually high rate of new HIV infections in the region requires urgency in better understanding treatment needs for People with Opioid Use Disorder who remain at the center of HIV transmission in the EECA region and for whom little gains have been made in HIV transmission and mortality [24].

Despite the important findings of validating a brief tool to guide addiction treatment in Russian-speaking people, there are limitations, including analysis of a convenience sample of patients voluntarily and consecutively enrolled on MOUD in a subset of regions throughout Ukraine, and the small size of the test–retest sample. This concern is tempered, however, given that the sample reflects the demographic characteristics of patients enrolled on MOUD from a sample of over 15,000 MOUD patients [20].

## Conclusion

The current version of the BASIS-24-R is statistically valid in Russian based on an initial validation using the steps of the Mapi method. It may be worth exploring an improved version of the scale in the area of relationships. Use of the BASIS-24-R has the potential to lead to the tailoring of MOUD programs to more closely align with other needs of those who enter care, therefore potentially resulting in improved MOUD admission and retention as well as improved quality of life as co-morbidities are addressed. Understanding the profile of those entering MOUD treatment may also help in the development of shared decision tools regarding MOUD treatment entry, as MOUD remains dramatically under-scaled in the face of the intertwined opioid, HIV and TB epidemics in the EECA region.

## Data Availability

The datasets used and/or analyzed during the current study are available from the corresponding author on reasonable request.

## Abbreviations

BASIS-24: Behavior and symptom identification scale
BASIS-24-R: Russian version of the BASIS-24
EECA: Eastern Europe and Central Asia
MOUD: Medications for opioid use disorder
PCA: Principal component analysis
PWID: People who inject drugs
